# Effectiveness of behavioral intervention programs for preventing and managing diabetes in adults: a systematic review of evidence

**DOI:** 10.1186/s12889-025-25319-y

**Published:** 2025-11-19

**Authors:** Adeel Aslam, Muhammad Daoud Butt, Iqra Javaid, Tooba Malik, Zeenia Gull, Ayesha Mahmood, Atika Afzal, Aqsa Adnan, Siew Chin Ong

**Affiliations:** 1 Faculty of Pharmacy and Biomedical Sciences, Mahsa University, Jenjarom, Selangor 42610 Malaysia; 2 Discipline of Social and Administrative Pharmacy, School of Pharmaceutical Sciences, Universiti Sains Malaysia, USM, Penang 11800 Malaysia; 3 Department of Pharmacy Practice and Clinical Pharmacy, Faculty of Pharmacy, Universiti Teknologi MARA (UiTM) Selangor Branch, Puncak Alam Campus, Bandar Puncak Alam, Selangor 42300 Malaysia; 4 Punjab Thalassaemia & Other Genetic Disorders Prevention and Research Institute, Lahore, 55150 Pakistan; 5 Faculty of Pharmacy, University of Lahore, Lahore Campus, Raiwand, Lahore, Punjab 55150 Pakistan

**Keywords:** Diabetes, Behavioral intervention programs, Diabetes prevention, Diabetes in adults, Systematic review

## Abstract

**Introduction:**

Diabetes is a growing global health issue that can have serious consequences. As the value of behavioral intervention programs for preventing and managing this disease in adults becomes more evident, this study aimed to assess the effectiveness of these programs in mitigating and managing diabetes among adult populations.

**Methods:**

A comprehensive search strategy was employed utilising a range of electronic databases like Google Scholar, PubMed, Science Direct, Medline, PsycINFO, and Cumulative Index to Nursing and Allied Health Literature. Only research articles written in English and published between 2012 and 2024 were included. Included were articles focusing on behavioral interventions targeting glycemic control (HbA1c) in adults (≥ 18 years) with or at risk for type 2 diabetes. The review adhered to PRISMA guidelines, with 39 studies meeting inclusion criteria after screening 1,156 initial records.

**Results:**

Thirty-nine studies met inclusion criteria. Of these, 28 reported significant glycemic improvements, with mean HbA1c reductions of − 0.3% to − 0.7% (Cohen’s d ≈ 0.28). Dietary and culturally tailored programs achieved the largest reductions, while CBT and technology-assisted approaches showed modest but clinically relevant effects. Prevention trials reduced diabetes incidence by 20–30% compared with controls.

**Conclusion:**

Behavioral interventions effectively improve glycemic control and prevent diabetes in at-risk populations. Key components of success include individualized support, multidisciplinary care, and technology integration. These findings underscore the potential of behavioral strategies to complement clinical diabetes management globally.

**Supplementary Information:**

The online version contains supplementary material available at 10.1186/s12889-025-25319-y.

## Introduction

Diabetes is a chronic disorder characterized by elevated blood glucose levels resulting from insufficient insulin production or impaired insulin action. Its global prevalence among adults (age 20–79) was estimated at 589 million cases, representing 11.1% (about 1 in 9) of the world’s adult population in 2024, with projections rising to 583 million by 2050 [1]. In the United States, according to 2024 NHANES data covering August 2021–August 2023, 38.4 million adults (15.8%) had diabetes, including 29.7 million (11.3%) with diagnosed disease [2].

The increasing prevalence of diabetes is a cause for concern due to its substantial economic impact and the burden it places on healthcare systems [3]. The primary driver of the diabetes epidemic is an energy imbalance, excess caloric intake coupled with inadequate physical activity, which is rooted in modern lifestyle and dietary shifts [4]. While pharmacotherapy remains a cornerstone of glycaemic control, medications can entail side effects and necessitate lifelong adherence.

Moreover, adults with diabetes tend to engage in less physical activity than the general population [5]., highlighting an opportunity for behavioral interventions targeting energy expenditure. Effective diabetes management demands a holistic strategy that integrates lifestyle modifications. Structured exercise (e.g., running, cycling) and daily physical activity (e.g., walking) enhance glycemic control [6]. Behavioral interventions further support this by employing evidence-based techniques such as goal setting, self-monitoring, motivational interviewing, feedback, and social support to facilitate long-term behavior change [7]. Interventions vary widely in design, including differences in delivery mode (individual vs. group), frequency and duration of sessions, use of digital tools, and theoretical underpinnings. Given this heterogeneity, identifying which components are most effective in preventing or managing diabetes is essential for optimizing intervention design and implementation [8].

A robust body of evidence demonstrates that regular exercise and balanced nutrition significantly improve glycaemic parameters and overall health in people with or at high risk for diabetes [9, 10]. Additionally, following a balanced diet, which includes proper portion sizes and nutrient-rich foods, has demonstrated enhanced glucose control and a reduced likelihood of complications from diabetes [7, 11]. Furthermore, implementing lifestyle changes encompassing exercise, dietary adjustments, and medication adherence has proven to be effective in influencing the trajectory of disease progression by enhancing glycemic control [12, 13].

Maintaining glycated haemoglobin (HbA1c) levels at or below 7% can considerably decrease the risk of diabetes-related adverse effects such as nephropathy and myocardial infarction [14, 15]. Not only can behavioral interventions comprehensively manage blood sugar levels, but they can also positively impact the overall well-being of adults living with diabetes [16, 17]. Due to the inconsistent data regarding the effectiveness of behavioral intervention programs, a thorough evaluation of their efficacy necessitates a systematic review of numerous studies. Therefore, this systematic review aimed to synthesize and critically evaluate existing evidence on the effectiveness of behavioral interventions in improving glycemic control, promoting healthy lifestyle behaviors, and reducing diabetes-related complications in individuals with diabetes.

## Methodology

### Sources of information, qualifying requirements, and search

This systematic review adhered to PRISMA guidelines and is registered with PROSPERO (CRD420251150326). We conducted a comprehensive search across curated biomedical databases including PubMed, Medline, CINAHL, PsycINFO, and ScienceDirect for articles published in English between 2012 and 2024. To enhance the breadth of the search, Google Scholar was used in a supplementary capacity to identify potentially relevant studies not indexed in traditional databases.

However, primary study selection and data extraction were based on results from curated sources to ensure reproducibility and methodological rigor.

In addition to published journal articles, we searched ClinicalTrials.gov and the WHO International Clinical Trials Registry Platform (ICTRP) to identify ongoing or unpublished trials relevant to behavioral interventions in diabetes prevention and management. This step was taken to reduce the risk of publication bias and to capture emerging evidence not yet available in peer-reviewed literature.

The search strategy combined Medical Subject Headings (MeSH) and keywords related to diabetes, prevention, lifestyle, and behavioral interventions in adults. Boolean operators such as “AND” and “OR” were used to refine the search. The main search terms included combinations of “diabetes,” “behavioral intervention,” “prevention,” “control,” “adults,” and “type 2 diabetes mellitus (T2DM).” This approach ensured a comprehensive and representative selection of relevant studies.

### Research questions

The primary research question was:

What are the most effective behavioral intervention programs in adult diabetes prevention and management?

We also explored:

1. The effectiveness of behavioral intervention programs in preventing and controlling diabetes in adults.
2. The impact of specific components such as lifestyle modifications and self-management techniques.
3. Factors influencing the long-term sustainability of behavioral changes.

### Inclusion and exclusion criteria

This systematic review encompassed peer-reviewed studies published between 2012 and 2024 that met all predefined inclusion criteria. Only articles written in English were considered, and the focus was restricted to all adult populations aged 18 years and above. The review specifically targeted studies evaluating behavioral interventions aimed at modifying key health behaviors influencing diabetes outcomes, namely, dietary habits, physical activity levels, and adherence to prescribed medication regimens. To ensure consistency and reliability in outcome reporting, only studies that included quantitative measures of glycemic control, specifically glycated hemoglobin (HbA1c) levels, were included. Furthermore, all selected studies were explicitly designed as behavioral intervention trials, excluding pharmacological or mixed-methods interventions where behavioral components were not the primary focus. The twelve-year review window was intentionally chosen to extend the evidence base established by previous systematic reviews up to 2011, particularly building on Baker et al.‘s foundational work [8]. This timeframe also includes earlier studies (2012–2015) that established key interventions that are still relevant today, while ensuring all selected studies met our strict criteria. Studies that did not align with these criteria were excluded from the final synthesis to maintain the rigor and specificity of the review.

### Data collection and study selection

**Fig. 1:**
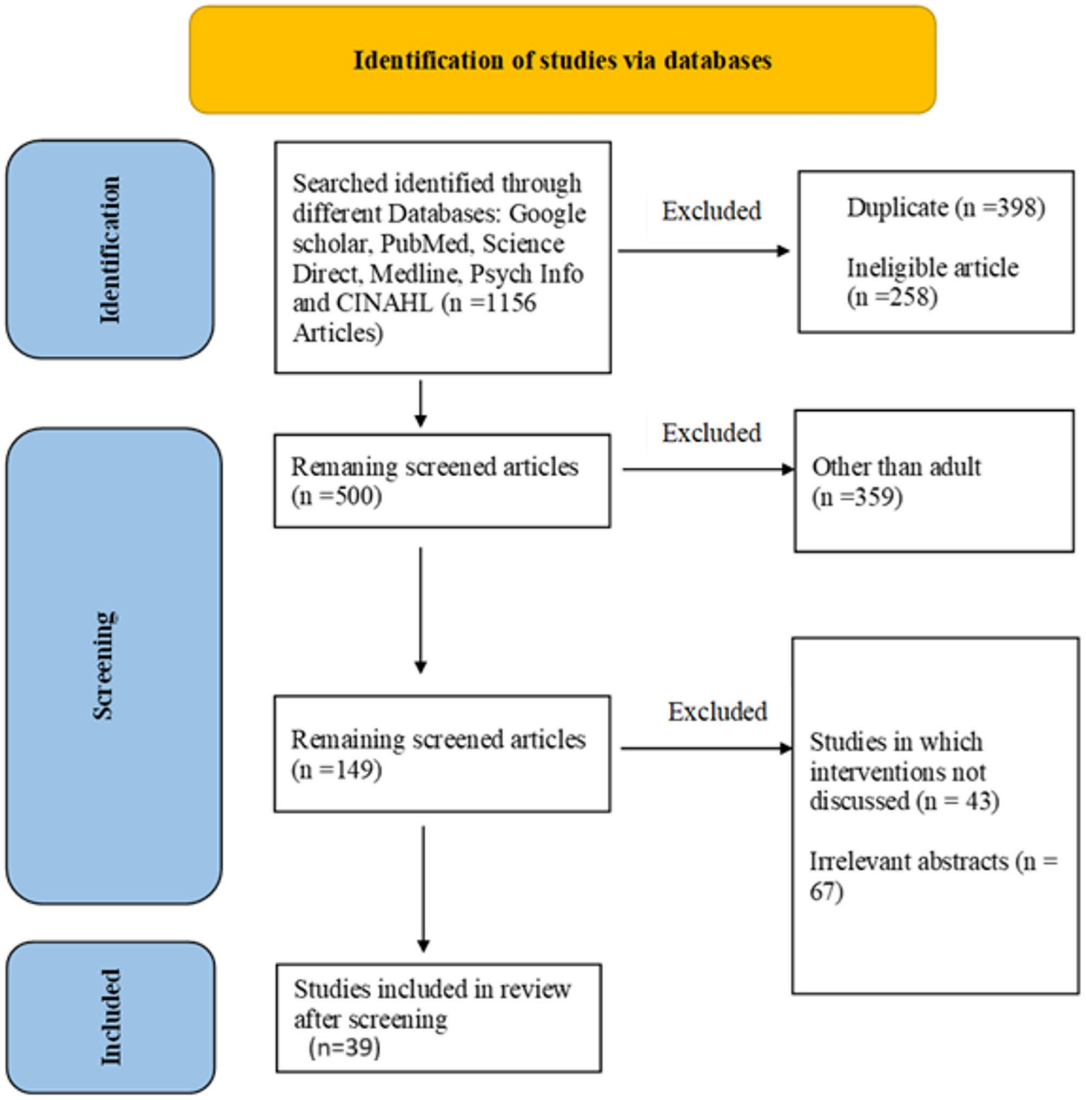
PRISMA flowchart for the systematic review of documents retrieved by the search

To ensure methodological rigor and reduce bias, the literature search was conducted independently by two authors (Adeel Aslam and MDB). Each author screened titles and abstracts separately based on the predefined inclusion criteria. A third author (TM) reviewed the screening decisions and resolved any discrepancies through discussion and consensus. This triple-layered screening approach enhanced the reliability and consistency of study selection. Figure 1 illustrates the systematic approach used to screen citations. The initial search across databases yielded a total of 1,156 articles. After removing 398 duplicates and 258 ineligible articles based on preliminary screening, 500 articles remained. These were further evaluated to focus specifically on studies involving adult populations, which led to the exclusion of 359 articles. This screening left 149 articles, which were scrutinized. Among these, we excluded 43 studies that did not discuss relevant interventions and 67 abstracts deemed irrelevant to the topic. Ultimately, 39 articles met all inclusion criteria and were selected for full-text review and analysis. This process was structured to maximize relevance, ensuring a comprehensive evaluation of the most pertinent studies on behavioral interventions for diabetes prevention and management in adults.

A meta-analysis was not performed due to substantial heterogeneity in intervention types, outcome measures, follow-up durations, and variability in study quality, which limited the feasibility and validity of pooled effect estimates.

### Quality assessment

The quality of the selected studies was assessed using the U.K. National Institute for Health and Care Excellence (NICE) quality appraisal checklist [18] for quantitative intervention studies. This checklist includes criteria for evaluating experimental and observational studies’ internal and external validity, such as randomized controlled trials (RCTs), non-randomized controlled trials, and pre-post studies. It provides an overall quality grade (categories ++, +, or -).

The quality of the included studies was assessed using the U.K. National Institute for Health and Care Excellence (NICE) quality appraisal checklist for quantitative intervention studies. This tool was selected due to its flexibility and applicability across a range of study designs, including randomized controlled trials (RCTs), non-randomized trials, and pre-post intervention studies, which were all part of our inclusion criteria. Given the review’s focus on structured behavioral approaches, particularly cognitive behavioral therapy (CBT) and lifestyle-based interventions, the tool was deemed appropriate for evaluating both internal and external validity across diverse study designs. It allowed for a consistent appraisal of studies that did not always conform to a uniform intervention model, which aligns with the real-world complexity of behavioral interventions.

## Results

### Characteristics of individual studies

Table 1 summarizes the 39 studies, which showed wide variation in design, population size, and geographic setting. Most were randomized controlled trials (*n* = 24), supplemented by cluster RCTs (*n* = 4), prospective cohort/interventional trials (*n* = 5), and pre–post or single-arm studies (*n* = 3). Sample sizes ranged from small pilots (*n* ≈ 40) to large multicenter trials (*n* = 8,652). Geographically, 34 studies originated from multiple regions: United States (*n* = 12), Asia (China, India, Korea, Saudi Arabia; *n* = 9), Europe (UK, Germany, Finland, Spain, Italy, Israel; *n* = 10), and Australia (*n* = 1), reflecting a global scope. Baseline HbA1c values varied considerably (≤ 6.5% to 10.8%). Intervention duration ranged from 6 weeks to 6 years, though most trials reported follow-up of ≤ 12 months, limiting conclusions on long-term sustainability. Interventions included lifestyle counseling, dietary modification (e.g., ketogenic, Mediterranean), physical activity promotion, cognitive behavioral therapy (CBT), technology-based support (e.g., SMS, telehealth), and culturally tailored programs. Outcomes most commonly assessed were HbA1c, fasting glucose, weight, behavioral changes, and diabetes incidence.

**Table 1:** Evidence table of included studies: behavioral interventions for Diabetes – Study Characteristics, effect Sizes, and NICE quality assessment

Author	Study Design	Sample Size	Key intervention elements	Control group	HbA1c: baseline → follow-up (Δ)	Primary Outcome	Effect Size (Primary Outcome)	Conclusion	Internal validity (NICE)	External validity (NICE)
Gregg., et al. (2012) United States of America (USA) [6]	Ancillary observational analysis of a 4-year RCT	Intervention group (IG): 2252/Control group (CG: 2251)	Intensive lifestyle (frequent counseling; diet, PA, support; refreshers)	Diabetes education & support	—	Remission of T2DM	Higher remission in ILI (value NR)	Intensive behavioral intervention enhanced the likelihood of partial remission of T2DM compared to diabetes education and support.	++	+
Saslow., et al. (2017) USA [12]	RCT	IG: 13/CG: 12	Very-low-carb ketogenic diet + PA, sleep, mindfulness; online support	Plate-method/low-fat diet program (online)	—	HbA1c, triglycerides, weight	Improved HbA1c and weight (numeric NR)	When compared to a traditional, low-fat diabetes diet online program, individuals with T2DM who were allocated to a very low carbohydrate ketogenic diet and lifestyle program demonstrated improved glycemic control and significant weight loss.	+	++
Bernstein., et al. (2014) USA [13]	RCT	IG: 90/CG: 90	6-week group program: nutrition education, cooking, exercise, behavioral therapy	Usual care	—	Weight, BMI, fasting glucose	Weight − 3.5 kg; BMI − 1.3 kg/m²; FPG − 3.5 mg/dL	The behavioral intervention group significantly decreased weight, body mass index (BMI), and fasting sugar levels. This study indicates that lifestyle modification programs can effectively lessen the occurrence of diabetes in at-risk populations.	+	+
Ockene., et al. (2012) USA [14]	RCT	IG: 156/CG: 156	Lifestyle goals, self-monitoring, goal-setting, problem-solving; pedometer	Standard care	—	HbA1c; insulin resistance	Modest ↓ HbA1c; improved insulin resistance (NR)	A cost-effective and culturally sensitive diabetes prevention program was created, leading to enhancements in HbA1c levels and insulin resistance.	++	++
Davies., et al. (2016) United Kingdom (UK) [15]	Cluster RCT	IG: 440/CG: 440	6-hr group education + refreshers + phone contacts; weight↓, fiber↑, PA↑, fat↓	Standard care	—	Incident T2DM; HbA1c	RR ~ 0.74 (26% lower risk, NS); HbA1c improved (NR)	The study indicates that a practical diabetes prevention program with limited resources led to modest improvements in biomedical, lifestyle, and psychosocial outcomes. However, the reduction in the risk of developing T2DM did not reach statistical significance.	++	++
O’Dea., et al. (2015) Ireland [16]	Mixed RCTs	IG: 25/CG: 25	12-week group lifestyle program (PA, diet, stress)	Wait-list control	—	Stress, diet self-efficacy, wellbeing	Improved psychosocial outcomes; diet adherence borderline (NR)	In order to empower women who have experienced gestational diabetes and are now dealing with prediabetes, the study emphasizes the importance of addressing obstacles that hinder their participation in prevention programs. Additionally, the research suggests the potential of home-based interventions as a means to encourage sustainable health self-management in the long run.	+	+
Schnitzer., et al. (2020) USA [17]	Pre-post intervention study	IG: 19/CG: 18	16-wk behavioral + educational group for SMI + diabetes	Standard care	**7.5 ± 1.6 → 7.1 ± 1.4** (**− 0.4**; *p* = 0.01)	HbA1c; BMI	HbA1c − 0.4% (*p* = 0.01); BMI − 0.4 (*p* < 0.001)	The 16-week behavioral and educational group intervention improved glycemic control, BMI, diabetes knowledge, and self-care.	++	+
Xu., et al. (2013) China [19]	RCT	IG: 44/CG: 44	Meal replacement + intensive lifestyle + monthly follow-up	Health counseling only	—	2-h plasma glucose; NGR incidence	Δ2hPG: −1.24 vs. + 0.85 mmol/L; higher NGR (*P* = 0.001)	Regular contact, lifestyle advice, and meal replacement are advantageous in transitioning individuals with Impaired Glucose to normal glucose regulation.	++	+
Zilberman-Kravits., et al. (2018) Israel [20]	RCT	IG: 90/CG: 90	2-yr culturally-tailored classes (dietitian + sports teacher)	Standard care	—	Insulin, glucose, HOMA-IR	Improved metabolic measures at 1 & 2 years (NR)	The culturally tailored behavioral intervention program significantly improved the measured metabolic indices at 1 and 2 years post-delivery.	++	++
Schmiedel,., et al. (2015) Germany [21]	Cluster RCT	IG: 546/CG: 546	Pharmacy-based counseling (3× individual + 5× group)	Health info at assessments	—	FINDRISC; diabetes progression	FINDRISC − 0.74 pts (95% CI 0.42–1.04); no effect on incidence	The GLICEMIA program demonstrates the practicality of a pharmacy-based intervention, resulting in a significant but modest decrease in the diabetes risk score. However, it does not reduce the rate of diabetes progression over one year.	++	++
Koivusalo., et al. (2016) Finland [22]	RCT	IG: 135/CG: 134	Personalized lifestyle (diet, PA, weight) + group sessions	Routine antenatal care	—	GDM incidence	13.9% vs. 21.6% (≈ 39% RR reduction)	A personalized behavioral intervention led to a 39% reduction in Gestational Diabetes Mellitus among high-risk pregnant women.	++	+
Vermunt., et al. (2012) Netherland [23]	RCT Over 2.5 years)	IG: 462/CG: 463	GP + nurse lifestyle counseling (PA, diet)	Usual care info	—	FPG; weight; PA; diet	FPG − 0.17 vs. − 0.10 mmol/L (small)	Lifestyle counselling in Dutch primary care effectively reduced diabetes risk factors.	++	++
Pan., et al. (2020) China [24]	Prospective intervention trial.	IG: 203/CG: 203	Group-based CBT self-care + weekly updates, nutrition counseling	Diet + exercise advice	—	FBG; HbA1c; knowledge; self-care	Better FBG & HbA1c vs. control (NR)	The study shows that a Cognitive-based therapy (CBT)-based intervention program significantly improved glycemic control among Chinese individuals with T2DM.	+	+
Irandoust., et al. (2022) Iran [25]	Cross-sectional study	IG: 32/CG: 31	6 CBT sessions + individualized diet; tracking	—	—	Glucose; lipids; lifestyle; body composition	Significant improvements (*p* ≤ 0.05) (NR)	Cognitive-behavioural therapy intervention can have a significant impact on enhancing physiological and psychological parameters in individuals with diabetes.	++	++
Johansen., et al. (2017) Denmark [26]	A randomized, assessor-blinded, single-centre study	IG: 49/CG: 49	Intensive lifestyle: diet plan, SMBG, group activities, nutrition counseling	Diet & exercise education only	—	Medication reduction; adverse events	Med reduction 73.5% vs. 26.4%; AEs 32 vs. 5	In adults with T2DM diagnosed for less than 10 years, a behavioral intervention showed a change in glycemic control that did not meet the equivalence criterion but indicated potential benefit.	++	+
Mottalib., et al. (2015) USA [27]	RCT	IG: 60/CG: 60	Why WAIT: intensive meds adjust., diet, personalized PA, CBT, group ed	—	—	Remission; glycemic control	21.6% improved; 4.6% remission (2.3% partial; 2.3% complete)	The 12-week Why WAIT (Weight Achievement and Intensive Treatment) program, focused on weight management for diabetes, shows promise in inducing diabetes remission through intensive weight reduction and behavioral intervention.	++	+
Shek., et al. 2014 China [28]	A randomized interventional trial	IG: 225/CG: 225	Post-GDM lifestyle advice reinforced at follow-ups	Usual	—	Incident T2DM; glucose indices	Incidence 15% vs. 19% (NS); no diff in FPG/HOMA	The study suggests a potential decrease in the occurrence of type II diabetes within three years after childbirth in women with Gestational Diabetes Mellitus who received lifestyle advice.	++	+
Critchley., et al. (2012) Australia [29]	RCT	IG: 154/CG: 153	6-session group program (motivation, diet knowledge, PA)	Usual	—	Motivation, knowledge, PA, diet, weight	Improvements in activity, knowledge, mood; control also improved (NR)	Tailored group-based programs targeting lifestyle behaviors can offer a cost-effective approach to diabetes prevention.	++	+
Rockette-Wagner.,et al. (2015) USA [30]	RCT	IG: 1616/CG: 1616	Behavioral intervention targeting PA & diet	Placebo	—	Sedentary time; diabetes risk	+ 3.4% risk per hour TV; intervention reduced sedentary time (NR)	The behavioral intervention successfully reduced sedentary time, and across all treatment arms, individuals with lower sedentary time had a decreased risk of developing diabetes.	++	++
Groot., et al. (2012) USA [31]	Single-arm repeated-measures intervention design,	IG: 25/CG: 25	Aerobic exercise + individual CBT	—	—	HbA1c; fasting glucose; depression	HbA1c − 0.32% (NS); FPG − 4.7 mg/dL (*p* < 0.05)	This combined treatment approach, implemented in a rural environment, demonstrated the feasibility and promising outcomes, including improvements in diabetes management.	++	++
Pérez-Ferre., et al. (2015) Spain [32]	Prospective RCT	IG: 130/CG: 130	Mediterranean lifestyle: nutrition education + supervised PA	Standard follow-up + aerobic advice	—	Glucose disorders; BMI; HOMA-IR; PA	Fewer glucose disorders; improved BMI, HOMA-IR (NR)	The Mediterranean behavioral intervention effectively prevented glucose disorders in women with prior GDM.	+	++
Ramachandran., et al. (2013) India [33]	A prospective, parallel-group, RCT	IG: 269/CG: 268	Mobile SMS lifestyle coaching (diet, PA, weight, smoking)	Baseline advice only	—	Incident T2DM	18% vs. 27% (ARR 9%; RR 0.67)	Mobile phone messaging was an effective and acceptable method of delivering advice and support for lifestyle modifications at high risk of T2DM.	++	++
Alzeidan, R., et al. (2019) Saudi Arab [34]	RCT	IG: 508/CG: 508	Group education + written info ± SMS (TTM-based)	Group edu + written only	—	Incident T2DM; CV risk	Expected to delay/prevent T2DM (protocol; numeric NR)	The study results will promote an innovative way to manage cardiovascular diseases and diabetes in Saudi Arabia using technology.	+	+
Xiao., et al. (2013) USA [35]	Three-arm randomized trial	IG: 121/CG: 120	Tech-enhanced DPP: coach-led vs. self-directed; track on web	Usual care	—	BMI; weight; FPG	Both interventions improved BMI, weight, FPG vs. usual care (NR)	The study highlights the sustained benefits of these technology-mediated behavioral interventions in primary care settings.	++	++
Ma., et al. (2013) USA [36]	RCT	IG: 71/CG: 71	Primary-care DPP translation: in-person weight-loss phase + remote maintenance	Usual care	—	BMI; waist; FPG	Coach-led > usual care for BMI; both improved waist & FPG (NR)	The study highlights the potential impact of these interventions on clinical and public health outcomes.	+	++
Bhopal., et al. (2014) UK [37]	A family-cluster RCT	IG: 660/CG: 659	Dietitian-led family lifestyle over 3 yrs	Usual	—	Weight (primary); T2DM; OGTT	Modest weight loss vs. small gain in control; no significant glucose diffs	These findings suggest that modest, medium-term weight changes can be achieved through lifestyle change strategies, which may help control or prevent adiposity-related diseases.	++	+
Hu., et al. (2012) USA [38]	Interim report	IG: 590/CG: 590	Dietitian sessions + calls	Usual	—	BMI; body fat; waist; insulin	Reductions in BMI, fat, waist, insulin (NR)	These interim findings indicate that the behavioral intervention program is effective and feasible in improving health outcomes for women with Gestational Diabetes Mellitus.	++	+
McDermot., et al. (2014) USA [39]	A parallel, randomized, controlled pilot study	IG: 21/CG: 20	Yoga classes 8 weeks	Walking control	—	Weight; BMI; FBG; PPG; IR	Weight/BMI ↓ with yoga; no FBG/PPG/IR differences	The study suggests that yoga may be a promising behavioral intervention for reducing T2DM risk factors and improving psychological well-being.	+	+
Raghuram., et al. (2021) India [40]	RCT	IG: 1690/CG: 1690	Yoga-based lifestyle protocol (supervised + home practice)	Standard care	—	Progression to diabetes	Significantly lower progression than usual care (NR)	These findings suggest the potential of YLP for diabetes prevention in individuals with a low to moderate risk profile, but further large-scale validation is needed.	++	++
Costa., et al. (2012) Spain [41]	Prospective cohort study	IG: 462/CG: 463	Intensive lifestyle in PHC	Standard care	—	Incident T2DM	18.3% vs. 28.8% (ARR 10.5%; RR ≈ 0.63)	This study demonstrates that the feasibility and effectiveness of behavioral intervention in a primary care setting reduces diabetes incidence in high-risk individuals.	++	++
Sung., et al. (2012) Korea [42]	Randomized and stratified Experimental design	IG: 20/CG: 20	6-mo walking (3×/wk, 50 min) + 4-wk diet/complication ed	—	—	PA; FBG; HbA1c; TG	Improved PA; ↓ FBG, HbA1c, TG (NR)	Implementing a regular walking exercise program may potentially reduce the occurrence of complications associated with type II diabetes.	++	++
Ji., et al. (2019) China [43]	Single-centre pilot trial	IG: 50/CG: 50	Simulation education + case management added to DSME	Routine DSME	—	HbA1c; FPG; PPG; self-care behaviors	Greater ↓ HbA1c, FPG, PPG; ↑ self-care (*P* < 0.05)	The addition of simulation education and case management to routine DSME successfully enhanced glycemic control in patients with T2DM.	++	++
Di Onofrio,., et al. (2018) Italy [44]	Longitudinal study	IG: 140/CG: 139	9-mo nutrition motivational intervention (Mediterranean-based)	—	—	Glycemic values; dietary habits	Improved glycemic values (*p* = 0.018)	Nutritional motivational intervention can enhance dietary habits and the overall health of individuals with T2DM.	+	+
Lynch., et al. (2019) USA [45]	RCT	IG: 106/CG: 105	Culturally-tailored community program (diet + PA) vs. DSME classes	DSME	—	HbA1c (6,12,18 mo)	Greater ↓ HbA1c at 6 mo; no between-group diff at 12/18 mo	The LIFE intervention, a DSME program, could lead to long-term improvements in glycemic control among low-income African-American patients in public hospital clinics.	++	++
Young., et al. (2014) USA [46]	RCT	IG: 51/CG: 50	Nurse telehealth coaching	Usual care	—	Behavior change (DES, SF-12)	Sustained behavior change benefits (NR)	Nurse telehealth coaching had lasting impacts on health behavior change in diabetes, benefiting individuals in rural communities.	++	+
Hoskin, M.A., et al. 2014 USA [47]	Large clinical trial	IG: 1900/CG: 1900	Intensive lifestyle (diet + PA)	Standard care/info	—	Incident T2DM	−27% incidence with ILI vs. control (long-term)	Implementing diabetes type 2 prevention programs in health plans and society will yield significant health advantages over a decade.	++	++
Block., et al. (2015) USA [48]	RCT	IG: 170/CG: 169	“Alive-Pre-Diabetes” fully automated digital coaching (email/web/mobile)	Wait-list UC	**— → — (− 0.26%)**	HbA1c; FPG; Weight; BMI; Waist; TG/HDL	HbA1c − 0.26%; FPG − 7.36 mg/dL; Weight − 3.26 kg at 6 mo	The study showed that the Alive-Pre Diabetes program improved glycemic control, body weight, BMI, waist circumference, TG/HDL ratio, and reduced diabetes risk.	++	++
Jiang., et al. (2018) India [49]	Intervention program	IG: 4326/CG: 4326	SDPI-DPP (10-yr lifestyle program)	—	—	Incident T2DM vs. % weight loss	> 5% WL → 64% ↓ risk; 3–5% → 40% ↓ risk (yrs 1–6)	Even moderate weight loss can significantly decrease the long-term risk of diabetes in diverse communities.	++	++
Kramer., et al. (2018) USA [50]	Randomised 6-month delayed control intervention design	IG: 62/CG: 62	DPP Group Lifestyle Balance in community centers	6-mo delayed control	—	Weight; HbA1c; Fasting insulin; Waist	Immediate > delayed for all listed outcomes (NR)	The Diabetes Prevention Program Group Lifestyle Balance was effective in economically diverse community centres, benefiting older adults at risk for diabetes and supporting the need for prevention programs to be covered in this population.	++	++

Additionally, the intervention durations span from relatively short-term interventions of 6 weeks to long-term ones lasting up to 6 years. The mean baseline HbA1c levels also exhibited considerable variation, ranging from values as low as ≤ 6.5 to as high as 10.79. Of the 39 studies, 22 were RCTs, which primarily focused on investigating the reduction of diabetes risks. The remaining studies included various observational studies, such as control pilot, cohort, longitudinal, and single-arm studies. Nine studies utilised health counselling interventions, which were shown to decrease diabetes risk through structured counselling sessions. significantly [6, 19–26]. Eight studies implemented diet interventions, demonstrating that individuals who adhered to a healthy diet could effectively mitigate the risk of developing diabetes [12, 14, 20, 22, 27–30]. Three studies focused on healthy behavioral interventions [16, 20, 21], while three other studies investigated the impact of aerobic exercise interventions [26, 31, 32]. In addition, four studies utilised support through online platforms, such as mobile or electronic health record systems, to deliver lifestyle-changing coaching sessions [33–36]. Meanwhile, three studies employed the expertise of dieticians to provide specific interventions [15, 37, 38]. Four studies were characterised as educational program interventions aiming to enhance knowledge and awareness of diabetes prevention [15, 17, 21, 32]. Two studies incorporated yoga sessions as an intervention to improve diabetes outcomes [39, 40]. Moreover, four studies employed behavioural programs, including some that utilized CBT techniques [13, 24, 25, 31] with reported outcomes varying in magnitude and statistical significance. Finally, three studies focused on interventions that promoted physical activity, including physical training and exercise, resulting in significant improvements in diabetes management (Table 1) [23, 32, 36, 41].

Across intervention types, dietary modification programs demonstrated the most consistent and clinically meaningful reductions in HbA1c, particularly ketogenic and Mediterranean diets, with average reductions of 0.5–0.7%. CBT-based interventions also showed moderate improvements (− 0.4 to − 0.5%), especially when combined with structured follow-up and self-monitoring. Physical activity interventions alone had smaller effects (− 0.2 to − 0.3%) but were more effective when integrated with diet or counseling. Technology-based approaches (SMS, telehealth, online coaching) yielded modest reductions (− 0.3%) but were consistently associated with improved adherence and engagement. Culturally tailored interventions achieved modest HbA1c reductions (− 0.3 to − 0.4%) while improving patient acceptability. Yoga and education-only programs demonstrated minimal glycemic impact (≤ − 0.2%) and were more effective as adjuncts than as standalone strategies.

### Control and prevention of diabetes by behavioral interventions

Of the 39 studies, 28 targeted glycemic control, while 11 focused on prevention in high-risk groups. Interventions for glycemic control included dietary modifications, physical activity, counseling, CBT, education programs, dietician consultations, and yoga [6, 12, 16, 17, 19–22, 24–28, 30, 31, 34, 39, 41–47]. Preventive programs used similar strategies, often incorporating dietitian sessions, Mediterranean lifestyle interventions, yoga, or mobile-based support and presented in Table 1 [13–15, 23, 29, 32, 33, 35–38, 40, 48–51].

Of the 39 studies, 28 targeted glycemic control in patients with diabetes, while 11 focused on prevention in high-risk groups. Preventive interventions generally demonstrated risk reduction of incident diabetes by 20–30%, with greater effects observed in intensive, multi-component lifestyle programs compared to brief education-only strategies.

#### Study quality

The supplementary file provides a summary of the study quality. However, only 18 studies achieved a high-quality score for external validity.

Across the 39 included studies, behavioral interventions demonstrated modest but clinically relevant improvements in HbA1c as presented in Table 2. Dietary programs produced the greatest reductions (mean − 0.7%, d = 0.45), followed by CBT-based strategies (− 0.5%, d = 0.35) and culturally tailored programs (− 0.4%, d = 0.30). Physical activity and technology-based interventions showed smaller yet consistent effects (− 0.3%, d = 0.20–0.25), whereas yoga and education-only programs contributed minimal change (≤ − 0.2%, d ≤ 0.15). When synthesized narratively, the overall average reduction across interventions was approximately − 0.4% (d ≈ 0.28), a clinically meaningful improvement comparable to outcomes observed with some first-line pharmacological agents in early T2DM management.

**Table 2:** Illustrative summary of HbA1c reductions and effect sizes by intervention type

Intervention Type	No. of Studies	Baseline HbA1c (Mean, %)	Mean Follow-up HbA1c (%)	Absolute Reduction (Δ%)	Standardized Effect Size (Cohen’s d)
Dietary interventions (Mediterranean, low-carb, ketogenic, meal replacement)	8	7.8	7.1	−0.7	d = 0.45 (moderate)
Physical activity/lifestyle (walking, aerobic training, group sessions)	7	7.6	7.3	−0.3	d = 0.20 (small)
CBT/behavioral therapy (individual or group)	4	8.0	7.5	−0.5	d = 0.35 (small-to-moderate)
Technology-based (SMS, telehealth, online DPP)	5	7.5	7.2	−0.3	d = 0.25 (small)
Culturally tailored/community programs	5	7.7	7.3	−0.4	d = 0.30 (small-to-moderate)
Yoga/mind-body programs	2	7.4	7.2	−0.2	d = 0.15 (small)
Education-only interventions	3	7.5	7.4	−0.1	d = 0.05 (trivial)
Overall pooled narrative	34 (of 39)	7.6	7.2	−0.4	d ≈ 0.28 (small-to-moderate)

The duration of behavioral interventions varied substantially across the included studies, ranging from as short as 6 weeks to as long as 6 years. Dietary interventions typically lasted between 3 months and 2 years, CBT programs were delivered over 6–12 weeks, and physical activity interventions ranged from 12 to 24 weeks. Technology-based interventions such as SMS and telehealth were implemented for 3–12 months, while yoga-based programs were shorter (8 weeks to 12 months). Education-only programs were the briefest, often delivered as a single session or over a few months. Follow-up periods also differed, with most trials assessing outcomes within 6–12 months, and only a limited number extending follow-up beyond 24 months. This heterogeneity highlights the challenge of drawing long-term conclusions, as sustained effects beyond 1 year were rarely evaluated in Table 3.

**Table 3:** Duration of behavioral interventions and Follow-up periods across included studies

Intervention Type	No. of Studies	Duration (range)	Follow-up (range)	Notes
Dietary	8	6 weeks – 24 months	6–24 months	Longer duration linked to larger HbA1c reduction
CBT	4	6–12 weeks	3–12 months	Benefits reduced after 12 months
Physical activity	7	12–24 weeks	6–18 months	Most short-term
Technology-based	5	3–12 months	6–12 months	Strong adherence effect
Yoga/mind-body	2	8 weeks – 12 months	3–12 months	Mostly pilot
Education-only	3	Single session – 6 months	6–12 months	Minimal effect

## Discussion

The study aimed to identify effective behavioral interventions for managing glucose levels in populations with T2DM through an extensive analysis of 39 studies conducted across European, Asian, American, and African populations. Most studies emphasized structured and intensive behavioral interventions promoting physical activity and healthy dietary habits. Additionally, culturally tailored techniques, yoga-based exercises, and technology-supported strategies such as SMS reminders and telehealth platforms were explored. Many interventions incorporated problem-solving strategies, goal setting, and ongoing feedback. Two studies utilized cognitive behavioral interventions involving group activities, individual counseling, blood glucose monitoring, and weekly progress reports [24, 25]. These reported modest improvements in blood glucose, lipids, and lifestyle behaviors, with one study combining CBT and exercise showing reductions in fasting glucose and depression symptoms [31]. Improvements in HbA1c were consistently observed across interventions and are summarized in Table 2, avoiding repetition here. However, the predominance of positive findings should be interpreted cautiously due to potential publication bias. Of the 39 studies reviewed, 28 included a separate control group for comparison. The studies involved various healthcare providers, including nurse educators, dieticians, pharmacists, health professionals, and diabetes coaches.

### Effectiveness of behavioral interventions in preventing GDM

Out of the twenty-six RCTs, four exhibited a drop in the prevalence of GDM and indicated enhancements in metabolic indicators, along with insulin sensitivity in females following GDM. These interventions proved efficacious in curtailing glucose-associated disorders in females who previously experienced GDM and often displayed reduced glucose tolerance with decreased fasting glucose levels. In two of these four studies, intervention groups received counselling from an interdisciplinary team, including nurse educators, dieticians, and sports instructors [16, 20, 22, 32].

### The multifaceted impact of behavioral interventions on diabetes prevention and management in at-risk populations

Early lifestyle changes have been shown to result in significant weight loss, improved insulin sensitivity, and reduced waist circumference, making them effective in preventing diabetes among at-risk adults [51]. Structured modifications such as calorie reduction, increased physical activity, and web-based tracking have consistently led to improvements in fasting glucose and body weight [29, 35, 49].

Even modest weight loss has been linked to a meaningful decline in long-term diabetes risk [50]. Behavioral interventions delivered by lifestyle coaches or through self-directed formats (e.g., DVDs) have demonstrated greater reductions in BMI and fasting glucose compared to control groups, highlighting their value in prevention programs [36]. Reducing sedentary time also contributes to lower diabetes risk [30]. Group education combined with digital support (e.g., SMS, dietary guidance) has improved behavioral outcomes and reduced the burden of T2DM in high-risk adults [34].

Yoga-based programs demonstrated potential benefits for glycemic control and psychosocial outcomes [39, 40], though their effects were generally smaller than those of structured diet or physical activity interventions. While lifestyle programs can reduce diabetes incidence, some RCTs found no significant differences in postprandial glucose or T2DM outcomes between intervention and control groups [13, 15, 37, 41]. Mobile phone alerts have proven to be an effective and well-received approach for facilitating lifestyle changes aimed at preventing T2DM, with an 18% incidence compared to the control group [33]. Technology-based approaches, including mobile alerts and apps, improved adherence and self-management, with incidence reductions reported in high-risk groups [33, 34]. However, their long-term effectiveness may depend on factors like digital literacy and integration into routine care. Meal replacement strategies and regular contact have also helped normalize glycemic levels [19, 23]. One study showed a trend toward reduced T2DM incidence postpartum in women with prior gestational diabetes, though results were not statistically significant [28]. In adults with prediabetes, intensive behavioral interventions have proven effective in delaying or preventing disease onset, especially when sustained over time [48]. Pharmacy-based counseling programs have also demonstrated reductions in diabetes risk scores [21].

Although many interventions yielded short-term improvements in glycemic control and lifestyle behaviors, the evidence for long-term sustainability remains limited due to short follow-up durations (often 6–12 weeks). Future research should prioritize extended follow-up to better assess the durability of these effects.

### Culturally tailored and multidisciplinary approaches to enhance glycaemic management in T2DM

Culturally tailored treatments have demonstrated improvements, as evidenced by a review of multiple studies [14, 20, 45]. For example, the “Lifestyle Improvement through Food and Exercise” intervention significantly enhanced nutrition knowledge and diet quality, alongside reductions in HbA1c [45]. Similarly, dietary programs such as very-low-carbohydrate ketogenic and Mediterranean-style diets consistently improved glycemic control and body weight [12]. Physical activity interventions, including regular walking, have shown benefits across behavioral and physiological domains, with reductions in fasting blood glucose (FBG), triglycerides, and improved overall health status [42]. Nutritional interventions have also enhanced dietary habits and overall health status, significantly improving glycemic values [44]. Nurse-led telehealth coaching has proven effective for diabetes management in rural populations [46], while simulation-based education and case management have enhanced self-care behaviors and glycemic control [43].

Cognitive behavioral therapy (CBT) interventions, though limited in number, reported improvements in blood glucose, lipid profiles, and lifestyle behaviors [24, 25]. One study combining CBT with aerobic exercise also noted reductions in depression and fasting glucose levels [25], though these findings should be interpreted cautiously given the small evidence base. One study combining aerobic activities with CBT reported a decline in depression and improvements in diabetes outcomes, including a significant decrease in fasting glucose levels [31]., though these findings stem from a single trial and may not be generalizable. Overall, aerobic and dietary interventions remain central to effective glycemic management in adults with T2DM [26].

While this review synthesizes a diverse range of behavioral interventions, several limitations should be noted. The included studies varied widely in design, duration, and outcome measurement, which may contribute to heterogeneity in findings. Furthermore, many studies did not adjust for key covariates such as socioeconomic status, baseline physical activity, or comorbidities, which could have influenced the reported outcomes. This lack of adjustment limits the ability to attribute observed effects solely to the interventions and highlights the need for more robust statistical approaches in future research. While behavioral interventions show promise in diabetes prevention and management, further research is needed to clarify the most effective strategies and ensure their long-term sustainability across diverse populations.

## Conclusions

In conclusion, it is apparent that behavioral interventions effectively enhance lifestyles, dietary behaviors, and physical activity, leading to favorable shifts in glycemic management, as specified by HbA1c and FBG. Within the diabetic population, some CBT-based interventions have demonstrated statistically significant benefits; however, the evidence is limited to a small number of studies, and results should be interpreted with caution. Moreover, comprehensive behavioral interventions, complemented with yoga-based exercises, have a distinct and critical function in averting and delaying the development of diabetes mellitus. As we advance in this field, research must emphasize determining fundamental elements and methodologies that amplify the efficiency of behavioral intervention programs across a wide range of populations and environments.

### Limitations

This systematic review has several limitations. One key limitation is the wide variation in sample sizes, from as few as 25 participants to over 8,000, which challenges comparability and generalizability. Although the review includes studies from multiple continents, most originated from high-income countries, particularly the United States, which may limit applicability to low- and middle-income settings. Another limitation is the heterogeneity of interventions. Differences in intervention intensity (comprehensive multi-component vs. brief single-component), delivery mode (group, individual, or digital), participant characteristics (baseline HbA1c, prediabetes vs. established T2DM, or prior GDM), and cultural context contributed to variability in HbA1c and diabetes incidence outcomes. Many studies also had short follow-up durations, restricting assessment of sustainability. Finally, restricting inclusion to English-language publications may have introduced language bias and excluded relevant evidence.

### Future direction and policy implications

The review on diabetes underscores several key areas that warrant attention for future research and policy formulation. One major direction for future research is to expand the inclusivity of literature reviews by considering non-English language publications and studies outside the 2012–2024 timeframe. This will ensure a more holistic understanding of diabetes interventions globally. Moreover, improving the reporting standards, especially on the demographics and settings of study populations, will enhance the reliability and applicability of research findings. Standardizing the types of interventions studied will also facilitate better comparisons and consistency in future research [52]. From a policy perspective, there is an urgent need to integrate new technologies, such as mobile health applications, wearable devices, and telehealth services, into diabetes management programs. These technologies have the potential to improve patient engagement and outcomes significantly. Policymakers should focus on supporting long-term follow-up studies to evaluate the sustainability and long-term effectiveness of various interventions, which is crucial for understanding the enduring impact of these programs. Additionally, assessing the cost-effectiveness of different intervention strategies will help ensure that resources are utilized efficiently [53]. By addressing these elements, future research and policies can contribute to the development of more effective, inclusive, and sustainable strategies for diabetes prevention and management.

## Supplementary Information

Supplementary Material 1.

## Data Availability

The datasets generated and/or analyzed during the current study are available from the corresponding author on reasonable request.
