# Supplementary material for: Effectiveness of behavioral intervention programs for preventing and managing diabetes in adults: a systematic review of evidence
Source: BMC Public Health. 2025 Nov 19;25:4052. doi: 10.1186/s12889-025-25319-y (PMC12628964; doi:10.1186/s12889-025-25319-y)
Supplement: Supplementary file 1 — Supplementary Material 1. [file 12889_2025_25319_MOESM1_ESM.docx]

| Sr. No | Checklist criteria | Gregg., et al.  (2012) | Saslow., et al  (2017) | Bernstein., et al.  (2014) | Ockene., et al.  (2012) | Davies., et al.  (2016) | O'Dea., et al.  (2015) | Schnitzer., et al  (2020) | Xu., et al.  (2013) | Zilberman-Kravits., et al.  (2018) | Schmiedel,., et al  (2015) | Koivusalo., et al.  (2016) | Vermunt., et al.  (2012) | Pan., et al.  (2020) | Irandoust., et al.  (2022) | Johansen., et al.  (2017) | Mottalib., et al.  (2015) | Shek., et al  2014 | Critchley., et al  (2012) | Rockette-Wagner.,et al.  (2015) | de Groot.,et al.  (2012) |
| --- | --- | --- | --- | --- | --- | --- | --- | --- | --- | --- | --- | --- | --- | --- | --- | --- | --- | --- | --- | --- | --- |
| 1.1 | Source population or area well described | ++ | ++ | ++ | ++ | ++ | ++ | ++ | ++ | ++ | ++ | ++ | ++ | ++ | ++ | ++ | ++ | ++ | ++ | ++ | ++ |
| 1.2 | Eligible population or area representative | ++ | + | + | ++ | ++ | ++ | + | ++ | + | ++ | + | ++ | + | ++ | + | + | + | ++ | ++ | ++ |
| 1.3 | Selected participants or area representative | + | + | ++ | ++ | ++ | + | + | + | + | ++ | + | ++ | + | ++ | + | ++ | + | + | ++ | + |
| 2.1 | Allocation: selection bias minimized | ++ | ++ | ++ | ++ | ++ | ++ | ++ | ++ | ++ | ++ | ++ | ++ | - | ++ | ++ | + | ++ | ++ | ++ | - |
| 2.2 | Interventions (& comparisons ) well described & appropriate | ++ | ++ | ++ | ++ | ++ | ++ | ++ | ++ | ++ | ++ | ++ | ++ | ++ | ++ | ++ | ++ | ++ | ++ | ++ | ++ |
| 2.3 | Allocation concealed | NR | + | + | + | NR | + | + | NR | NR | ++ | ++ | + | - | NR | ++ | + | NR | NR | NR | + |
| 2.4 | Participants &/or investigators blinded | NR | - | - | + | NR | - | NR | NR | NR | + | - | NR | - | - | + | NR | NR | NR | + | - |
| 2.5 | Exposure to intervention & comparison adequate | ++ | ++ | ++ | ++ | ++ | ++ | ++ | ++ | ++ | ++ | ++ | ++ | ++ | ++ | ++ | ++ | ++ | ++ | ++ | ++ |
| 2.6 | Contamination acceptably low | + | + | + | ++ | ++ | NR | NR | NR | + | ++ | + | NR | + | ++ | + | NR | + | + | NR | - |
| 2.7 | Other interventions similar in groups | + | ++ | ++ | ++ | ++ | + | + | ++ | + | ++ | + | ++ | + | ++ | ++ | + | + | + | + | + |
| 2.8 | All participants accounted for at study's conclusion | + | + | ++ | ++ | + | NR | + | ++ | + | ++ | + | ++ | NR | ++ | NR | + | + | ++ | ++ | ++ |
| 2.9 | Setting reflects usual UK practice | - | + | + | - | + | - | ++ | + | + | + | ++ | + | + | ++ | - | + | ++ | ++ | + | ++ |
| 2.10 | Intervention or control reflects usual UK practice | - | ++ | + | - | + | + | + | ++ | ++ | + | ++ | + | + | + | - | ++ | + | + | ++ | + |
| 3.1 | Outcome measures reliable | ++ | + | + | ++ | ++ | + | ++ | ++ | ++ | ++ | ++ | ++ | ++ | ++ | + | ++ | ++ | ++ | ++ | ++ |
| 3.2 | Outcome measures complete | ++ | ++ | ++ | ++ | ++ | + | + | ++ | ++ | ++ | + | ++ | + | ++ | ++ | ++ | ++ | + | + | ++ |
| 3.3 | All-important outcomes assessed | ++ | ++ | ++ | ++ | ++ | ++ | + | ++ | ++ | ++ | ++ | ++ | ++ | ++ | ++ | + | + | + | ++ | ++ |
| 3.4 | Outcomes relevant | ++ | ++ | ++ | ++ | ++ | + | ++ | ++ | ++ | ++ | NR | ++ | + | ++ | ++ | ++ | ++ | ++ | + | + |
| 3.5 | Similar follow-up times in groups | + | - | ++ | ++ | ++ | ++ | + | ++ | ++ | ++ | + | ++ | NR | ++ | ++ | ++ | ++ | + | ++ | + |
| 3.6 | Follow-up time meaningful | ++ | + | + | ++ | ++ | ++ | + | ++ | ++ | ++ | + | ++ | + | ++ | + | ++ | ++ | + | ++ | + |
| 4.1 | Groups similar at baseline | ++ | ++ | ++ | ++ | ++ | + | ++ | + | ++ | ++ | ++ | ++ | + | ++ | ++ | ++ | ++ | + | + | NA |
| 4.2 | ITT analysis conducted | + | + | NR | ++ | NR | ++ | + | ++ | + | ++ | ++ | ++ | ++ | NR | ++ | + | ++ | ++ | + | ++ |
| 4.3 | Study sufficiently powered | ++ | - | - | + | + | ++ | + | + | ++ | NR | NR | NR | + | + | + | + | + | + | NR | + |
| 4.4 | Estimates of effect size given or calculable | ++ | ++ | + | ++ | ++ | ++ | + | ++ | ++ | ++ | ++ | ++ | ++ | ++ | ++ | ++ | ++ | ++ | ++ | ++ |
| 4.5 | Analytical method appropriate | ++ | ++ | - | + | ++ | + | ++ | ++ | ++ | ++ | ++ | ++ | ++ | ++ | ++ | ++ | ++ | ++ | ++ | ++ |
| 4.6 | Precision of intervention effects given or calculable | ++ | + | + | ++ | ++ | + | ++ | + | ++ | ++ | ++ | ++ | + | ++ | + | + | + | + | + | ++ |
| 5.1 | Study results internally valid (i.e. unbiased) | ++ | + | + | ++ | ++ | + | ++ | ++ | ++ | ++ | ++ | ++ | + | ++ | ++ | ++ | ++ | + | + | ++ |
| 5.2 | Findings generalizable to source population (i.e. externally valid) | + | ++ | + | ++ | ++ | + | ++ | + | ++ | ++ | + | ++ | + | ++ | ++ | + | + | ++ | + | + |

| Sr. No | Checklist criteria | Pérez-Ferre., et al.  (2015) | Ramachandran., et al.  (2013) | Alzeidan, R.,et al.  (2019) | Xiao., et al.  (2013) | Ma., et al.  (2013) | Bhopal., et al.  (2014) | Hu., et al.  (2012) | McDermot., et al.  (2014) | Raghuram., et al.  (2021) | Costa., et al.  (2012) | Sung., et al.  (2012) | Ji., et al  (2019) | Di Onofrio,., et al.  (2018) | Lynch., et al.  (2019) | Young., et al.  (2014) | Hoskin, M.A., et al.  2014 | Block., et al  (2015) | Jiang., et al.  (2018) | Kramer., et al.  (2018) |
| --- | --- | --- | --- | --- | --- | --- | --- | --- | --- | --- | --- | --- | --- | --- | --- | --- | --- | --- | --- | --- |
| 1.1 | Source population or area well described | ++ | ++ | ++ | ++ | ++ | ++ | ++ | ++ | ++ | ++ | ++ | ++ | ++ | ++ | ++ | ++ | ++ | ++ | ++ |
| 1.2 | Eligible population or area representative | ++ | + | ++ | ++ | + | + | ++ | + | + | ++ | ++ | + | + | + | + | ++ | + | + | + |
| 1.3 | Selected participants or area representative | ++ | + | + | + | + | + | ++ | + | + | + | ++ | ++ | ++ | ++ | ++ | ++ | ++ | + | + |
| 2.1 | Allocation: selection bias minimized | ++ | ++ | ++ | ++ | ++ | ++ | ++ | ++ | ++ | + | ++ | + | - | ++ | ++ | ++ | ++ | + | ++ |
| 2.2 | Interventions (& comparisons ) well described & appropriate | ++ | ++ | ++ | ++ | ++ | ++ | ++ | ++ | ++ | ++ | + | ++ | ++ | ++ | ++ | ++ | ++ | ++ | ++ |
| 2.3 | Allocation concealed | NR | ++ | ++ | + | + | ++ | + | ++ | + | - | + | + | - | - | + | NR | ++ | NR | - |
| 2.4 | Participants &/or investigators blinded | NR | - | ++ | + | + | + | - | - | - | - | - | + | - | + | - | - | + | NR | - |
| 2.5 | Exposure to intervention & comparison adequate | ++ | ++ | ++ | ++ | ++ | ++ | ++ | ++ | ++ | ++ | NA | ++ | ++ | ++ | ++ | ++ | ++ | ++ | ++ |
| 2.6 | Contamination acceptably low | ++ | ++ | ++ | + | + | ++ | ++ | + | ++ | + | + | + | + | - | + | + | + | + | + |
| 2.7 | Other interventions similar in groups | ++ | ++ | ++ | + | ++ | ++ | ++ | ++ | ++ | ++ | NA | ++ | + | ++ | + | ++ | + | ++ | ++ |
| 2.8 | All participants accounted for at study's conclusion | ++ | + | + | ++ | ++ | ++ | ++ | ++ | NR | + | ++ | + | ++ | + | + | ++ | + | ++ | + |
| 2.9 | Setting reflects usual UK practice | ++ | + | ++ | ++ | ++ | ++ | + | ++ | + | ++ | + | ++ | ++ | + | ++ | ++ | ++ | + | + |
| 2.10 | Intervention or control reflects usual UK practice | ++ | + | ++ | + | + | ++ | ++ | + | ++ | + | ++ | ++ | ++ | ++ | + | ++ | + | ++ | + |
| 3.1 | Outcome measures reliable | + | ++ | + | ++ | ++ | + | ++ | + | ++ | ++ | ++ | ++ | + | ++ | + | + | ++ | ++ | ++ |
| 3.2 | Outcome measures complete | + | + | + | + | + | ++ | ++ | + | ++ | + | ++ | ++ | + | ++ | + | + | ++ | + | ++ |
| 3.3 | All-important outcomes assessed | ++ | ++ | + | ++ | ++ | ++ | ++ | + | ++ | ++ | ++ | ++ | + | ++ | ++ | ++ | ++ | ++ | ++ |
| 3.4 | Outcomes relevant | ++ | ++ | NR | ++ | + | ++ | ++ | ++ | ++ | + | ++ | ++ | + | ++ | ++ | ++ | ++ | ++ | ++ |
| 3.5 | Similar follow-up times in groups | ++ | ++ | + | ++ | NR | ++ | ++ | + | ++ | + | ++ | ++ | + | ++ | + | ++ | ++ | ++ | ++ |
| 3.6 | Follow-up time meaningful | ++ | ++ | + | ++ | ++ | ++ | ++ | + | ++ | ++ | ++ | ++ | + | ++ | + | ++ | ++ | ++ | ++ |
| 4.1 | Groups similar at baseline | ++ | ++ | NR | + | + | ++ | ++ | ++ | ++ | ++ | ++ | ++ | + | + | + | ++ | ++ | ++ | ++ |
| 4.2 | ITT analysis conducted | ++ | ++ | - | + | ++ | + | NR | ++ | ++ | + | ++ | NR | - | ++ | ++ | ++ | ++ | ++ | ++ |
| 4.3 | Study sufficiently powered | ++ | ++ | NR | NR | ++ | ++ | NR | - | + | ++ | ++ | NR | - | + | + | ++ | ++ | ++ | ++ |
| 4.4 | Estimates of effect size given or calculable | ++ | ++ | - | ++ | ++ | ++ | ++ | + | ++ | ++ | ++ | ++ | + | ++ | + | ++ | ++ | ++ | ++ |
| 4.5 | Analytical method appropriate | ++ | ++ | + | ++ | ++ | ++ | ++ | ++ | ++ | ++ | ++ | ++ | + | ++ | ++ | ++ | ++ | ++ | ++ |
| 4.6 | Precision of intervention effects given or calculable | ++ | ++ | + | + | ++ | ++ | ++ | + | ++ | ++ | ++ | ++ | + | ++ | + | ++ | ++ | ++ | ++ |
| 5.1 | Study results internally valid (i.e. unbiased) | ++ | ++ | + | ++ | + | ++ | ++ | + | ++ | + | ++ | ++ | + | ++ | ++ | ++ | ++ | ++ | ++ |
| 5.2 | Findings generalizable to source population (i.e. externally valid) | ++ | ++ | + | + | + | ++ | ++ | + | ++ | + | ++ | + | + | ++ | + | ++ | ++ | ++ | + |

- ++ Indicates that for that particular aspect of study design, the study has been designed/conducted in such a way as to minimise the risk of bias.
- + Indicates that either the answer to the checklist question is not clear from the way the study is reported, or that the study may not have addressed all potential sources of bias for that particular aspect of study design.
  - - Reserved for those aspects of the study design in which significant sources of bias may persist.
- Not reported (NR) Reserved for those aspects in which the study under review fails to report how they have/might have been considered.
- Not applicable (NA) Reserved for those study design aspects which are not applicable given the study design under review
